# Bone mineral density among children living with HIV failing first-line anti-retroviral therapy in Uganda: A sub-study of the CHAPAS-4 trial

**DOI:** 10.1371/journal.pone.0288877

**Published:** 2023-07-20

**Authors:** Eva Natukunda, Alex Szubert, Caroline Otike, Imerida Namyalo, Esther Nambi, Alasdair Bamford, Katja Doerholt, Diana M. Gibb, Victor Musiime, Phillipa Musoke

**Affiliations:** 1 Joint Clinical Research Centre, Kampala, Uganda; 2 Medical Research Council Clinical Trials Unit at University College London, London, United Kingdom; 3 Department of Paediatrics, College of Health Sciences, Makerere University Kampala, Kampala, Uganda; 4 Makerere University-Johns Hopkins University Research Collaboration (MUJHU CARE), Kampala, Uganda; Nigerian Institute of Medical Research, NIGERIA

## Abstract

**Background:**

Children living with perinatally acquired HIV (CLWH) survive into adulthood on antiretroviral therapy (ART). HIV, ART, and malnutrition can all lead to low bone mineral density (BMD). Few studies have described bone health among CLWH in Sub-Saharan Africa. We determined the prevalence and factors associated with low BMD among CLWH switching to second-line ART in the CHAPAS-4 trial (ISRCTN22964075) in Uganda.

**Methods:**

BMD was determined using dual-energy X-ray Absorptiometry (DXA). BMD Z-scores were adjusted for age, sex, height and race. Demographic characteristics were summarized using median interquartile range (IQR) for continuous variables and proportions for categorical variables. Logistic regression was used to determine the associations between each variable and low BMD.

**Results:**

A total of 159 children were enrolled (50% male) with median age (IQR) 10 (7–12) years, median duration of first -line ART 5.2(3.3–6.8) years; CD4 count 774 (528–1083) cells/mm^3^, weight—for–age Z-score -1.36 (-2.19, -0.65) and body mass index Z-score (BMIZ) -1.31 (-2.06, -0.6). Low (Z-score≤ -2) total body less head (TBLH) BMD was observed in 28 (18%) children, 21(13%) had low lumbar spine (LS) BMD, and15 (9%) had both. Low TBLH BMD was associated with increasing age (adjusted odds ratio [aOR] 1.37; 95% CI: 1.13–1.65, p = 0.001), female sex (aOR: 3.8; 95% CL: 1.31–10.81, p = 0.014), low BMI (aOR 0.36:95% CI: 0.21–0.61, p<0.001), and first-line zidovudine exposure (aOR: 3.68; 95% CI: 1.25–10.8, p = 0.018). CD4 count, viral load and first- line ART duration were not associated with TBLH BMD. Low LS BMD was associated with increasing age (aOR 1.42; 95% CI: 1.16–1.74, p = 0.001) and female sex: (aOR 3.41; 95% CI: 1.18–9.8, p = 0.023).

**Conclusion:**

Nearly 20% CLWH failing first-line ART had low BMD which was associated with female sex, older age, first-line ZDV exposure, and low BMI. Prevention, monitoring, and implications following transition to adult care should be prioritized to identify poor bone health in HIV+adolescents entering adulthood.

## Introduction

Globally, 38.4 million people are living with HIV (PLWH), with 1.7 million children aged 0–14 years. There were 160,000 new paediatric infections in 2021 with 90% living in Sub-Saharan Africa. Among children living with HIV (CLWH), 52% have access to antiretroviral therapy (ART) [1]. The World Health Organization (WHO) recommends that ART should be is initiated as soon as the HIV diagnosis is made in infants, children and young people [2]. Children now live longer into adulthood because of early ART initiation hence, the relative impact of long-term HIV and ART- related comorbidities, such as low bone mineral density (BMD) is becoming much more relevant. Low childhood BMD may result in suboptimal peak bone mass in adulthood and may predispose to osteoporosis and fracture risk in adulthood [3].

Low BMD has been previously described in PLWH and CLWH but most studies have been conducted in high-income countries [4, 5]. The prevalence of low BMD ranges from 20% to 80% in adults living with HIV [6, 7], and 4–32% in CLWH [8–10]. Few studies have been conducted in resource-limited settings where the majority of CLWH reside and where malnutrition, which also contributes to low BMD, is common [8, 10–12].

The aetiology of low BMD in the context of HIV infection is multifactorial. Relevant factors include uncontrolled HIV with a high viral load which favors osteoclast recruitment, low Vitamin D levels, low body mass index (BMI) and physical inactivity [13, 14]. Some antiretroviral drugs, including protease inhibitors, tenofovir disoproxil fumarate (TDF), and zidovudine (ZDV) contribute to low BMD while nevirapine (NVP) may be protective [8, 13]. Initiation of ART in treatment naïve adults or switching treatment due to virologic failure has also been found to be associated with reduced BMD in the first year followed by stabilization [10, 11, 13].

Bone health has not been widely studied in resource-limited settings yet it has the potential to be a long-term complication in PLWH. Under recognition is in part due to limited access to diagnostic dual-energy X-ray absorptiometry (DXA) facilities that detect low BMD before fracture occurrence.

In this sub-study of the CHAPAS-4 trial, we describe the baseline characteristics of a cohort of patients enrolled in the toxicity sub-study of the CHAPAS 4 trial in addition to reporting the bone health status of children switching to second-line ART at enrolment. We also examined the prevalence and risk factors associated with low BMD among children switching to second-line ART.

## Materials and methods

We conducted a cross-sectional study using baseline data from the toxicity sub-study of the CHAPAS 4 clinical trial, an ongoing open-label trial evaluating the virologic response to alternative second line antiretroviral therapy in CLWH.

In CHAPAS 4, 919 children aged 3–15 years failing on their 2NRTI+NNRTI- based first-line regimen with a viral load of 400copies/ml or more were randomized to receive tenofovir alafenamide/emtricitabine (TAF/FTC) or standard of care (abacavir/lamivudine [ABC/3TC] or zidovudine/lamivudine [ZDV/3TC], depending on the first-line treatment. Children were also randomized in a factorial design to dolutegravir (DTG) or darunavir/ritonavir (DRV/r) or atazanavir/ritonavir (ATV/r) or lopinavir/ritonavir (LPV/r). They are being followed for 96 weeks. In the toxicity sub-study, 444 children from study sites in Zimbabwe and Uganda were simultaneously enrolled to evaluate the potential renal and bone effects of TAF. Of these, 159 participants aged 5 years and above were enrolled at Joint Clinical Research Centre (JCRC) and consented to a DXA scan. The Centre recruited study participants from the paediatric clinic in the facility and neighbouring public health facilities in Wakiso, Mpigi, and Mukono districts. Reference BMD Z-scores were available for children who were ≥ 5 years and hence children who were 5 years and above were included for this study [15].

### Measurements

Data were collected at enrolment into the CHAPAS 4 trial from January 2019 to March 2021. A case report form was used to collect sociodemographic and clinical data. These included sex, age, and WHO classification of HIV disease [16], first-line ART and duration on treatment. Weight was measured using a Seca®weight scale, height was measured by wall mounted Seca® 206 stadiometer. BMI was calculated as weight (kg)/height (m^2^). Weight, height and BMI Z-scores were measured using the British 1990 reference data [17]. Blood samples were collected to determine the viral load and CD4 cell counts by real-time HIV-RNA (COBAS Ampliprep/Taqman 96 analyzer-detection range of 20–10,000,000 copies per ml) and BD FACS Calibur respectively.

BMD was measured using one DXA scan (Hologic Discovery Wi Apex 13.5, Hologic Bedford, MA USA) for all participants. DXA scans were conducted by an experienced technician while the participant was lying still and flat on an examination table. A posterior–anterior scan was obtained for four lumbar spines (L1-L4) and the whole body in the array mode. The lumbar spine (LS) BMD assesses trabecular bone density while the whole- body is largely cortical. The head was excluded from the analysis as recommended by International Society for Clinical Densitometry 2019 (ISCD) [18]. BMD was measured in grams per square centimetre. Normative values for sex, age, race and height adjusted Z-scores were obtained from bone mineral density children study (BMDCS) reference data [15]. Low BMD was defined as a LS or total body less head (TBLH) BMD Z-score of less than or equal to -2 according to the ISCD [18].

### Quality control

A standard operating procedure was developed for participant preparation for BMD determination. The DXA scanner was calibrated by the technician daily for accuracy of the spine BMD determination with a phantom scan, standardization of the whole-body BMD determination was performed annually and a uniformity test was performed weekly.

### Ethical review

The main CHAPAS 4 trial was approved by the JCRC institutional review board (reference–JC 1417).

Caretakers of the enrolled children gave written informed consent; children aged eight years and above gave written informed assent as appropriate and according to the knowledge of their HIV status.

### Statistical analysis

Data were exported to STATA (StataCorp Texas USA) for analysis. Participant characteristics were summarized using median with interquartile range (IQR) for numerical data. Categorical data were summarized using frequencies and proportions. Low BMD was defined as either a height adjusted TBLH BMD or LS BMD z-score less than or equal to -2.

To measure the association of low BMD with other exposure variables, a logistic regression model was used. Bivariate analysis was performed for each of the independent variables to determine whether they were independently associated with low BMD using odds ratios and p-values. In multivariate analysis, stepwise backward elimination method was used to identify the independent variables with P-values <0.05 which were considered statistically significant. Confounding was assessed for all the non-significant independent variables and a relative difference of 10% or more between the crude and adjusted OR was considered as confounding.

## Results

A total of 159 CLWH failing their first-line ART regimen were enrolled. The median age was 10.0 years (IQR 7.0, 12.0) and 79/159 (49%) participants were male. The median BMI z-score was -1.31 (IQR-2.06, -0.60). The median age at ART initiation and duration of first-line ART were 4.0 (IQR 2.0, 7.0) and 5.2 (IQR 3.3, 6.8) years, respectively. The median CD4 count was 774 (528, 1083) cells/ul and all children had viral loads ≥400 copies/ml, as per the main trial inclusion criteria. The majority of the participants were stable: 144 (90.6%) had WHO clinical stage 1 and 2, with only 15 (9.45%) ever having stage 3 and 4 (**Table 1**).

**Table 1:** Characteristics of study participants, N = 159.

Characteristic	Measure
Age: median (IQR)	10 (7,12)
Male sex (n/%)	79 (49.7)
**Anthropometry**	
Weight-for-age-Z-score (WAZ): median (IQR)	-1.36 (-2.19, -0.65)
Height-for-age-Z-score (HAZ): median (IQR)	-1.12 (-1.58, -0.10)
BMI- for- age-Z-score: median (IQR)	-1.31 (-2.06, -0.60)
BMI- for- age-Z-score:<-2 (%)	18 (11.3)
**HIV characteristics**	
CD4 count (cells/ul): median (IQR)	774 (528, 1083)
CD4%, median (IQR)	31 (22, 37)
Viral load (log_10_copies/ml): median (IQR)	4.25 (3.73, 4.85)
**WHO Stage (n/N)**	
Stage 1&2	144 (90.6%)
Stage 3&4	15 (9.4%)
**First- line ART regimen (n/N)**	
ABC- based	75 (47.2%)
ZDV- based	83 (52.2%)
EFV- based	80 (50.3%)
NVP- based	79 (49.7%)
Age at First-line ART initiation: median (IQR)	4 (2,7)
**First- line ART duration in years**: median (IQR)	5.21 (3.33, 6.84)
**Bone mineral density measures**	
**Lumbar spine**	
BMD g/cm^3^: median (IQR)	0.545 (0.487–0.648)
LSBMD_HAZ:_ median (IQR)	-0.99 (-1.46, -0.34)
Low lumbar BMD n/N (%)	21 (13.2)
**TBLH**	
BMDg/cm^3^: median (IQR)	0.656 (0.577, 0.732)
TBLH _HAZ:_ median (IQR)	-1.37 (-1.87, -0.81)
Low TBLH BMD n/N (%)	28 (17.6)
Low LSBMD with low TBLH BMD	15 (9.4)

Abbreviations: IQR = interquartile range, BMI = body mass index, LSBMD = lumbar spine bone mineral density, TBLH BMD = total body less head bone mineral density, HAZ = height adjusted Z- score; ZDV, zidovudine; NVP = nevirapine; EFV = efavirenz, ABC = abacavir

Associations with low TBLH BMD are summarised in **Table 2**. In the bivariate analysis, participants exposed to ZDV-based first-line therapy were more likely to have low TBLH BMD compared to those on ABC crude OR [cOR] = 3.2, p = 0.012). Increasing age (cOR = 1.33 per year older p < 0.001) and BMI (cOR = 0.43 per unit increase, p<0.001) were associated with low TBLH BMD. There was no significant association between low TBLH BMD and absolute CD4 count, viral load, time on first- line ART or height.

**Table 2:** Bivariate total body less head BMD analysis, N = 159.

Characteristic	Abnormal BMD	Normal BMD	cOR	95%CI	P-Value
	N (%)	N (%)			
**Sex**					
Male	10 (12.7)	69 (87.3)	1		
Female	18 (22.5)	62 (77.5)	2.00	0.86–4.7	0.107
**WHO stage**					
Stage 1&2	24 (16.7)	120 (83.3)	1		
Stage 3&4	4 (26.7)	11 (73.3)	1.82	0.53–6.19	0.339
**Regimen**					
ABC- based	7 (9.3)	68 (90.7)	1		
ZDV- based	21 (25.0)	63 (75.0)	3.2	1.29–8.14	0.012
EFV- based	14 (17.5)	66 (82.5)	1		
NVP- based	14 (17.7)	65 (82.3)	1.01	0.45–2.29	0.97
	**Median (IQR)**	**Median (IQR)**			
Age	12 (10,14)	9 (7,12)	1.33	1.13–1.57	0.001
BMI-Z	-2.0 (-3.0, -1.0)	-1.0 (-2.0, -1.0)	0.43	0.28–0.66	<0.001
Abs CD4 count	738 (564.5,1052)	774 (524,1083)	1.00	0.99–1.00	0.953
CD4%	31 (23,35.5)	30 (22,37)	1.00	0.96–1.03	0.948
HIV RNA log _10_ copies per ml	4.33 (3.87,4.94)	4.24 (3.73,4.84)	1.05	0.62–1.78	0.86
Time on first-line ART	6.0 (4.0,7.0)	5.0 (3.0,7.0)	1.06	0.91–1.25	0.439
Age at first-line ART start	6.0 (4.0,8.0)	4.0 (2.0,6.0)	1.22	1.06–1.399	0.05

Abbreviations: IQR = interquartile range, BMI = body mass index, LSBMD = lumbar spine bone mineral density, TBLH BMD = total body less head bone mineral density, HAZ = height adjusted Z- score; ZDV = zidovudine, EFV = efavirenz, NVP = nevirapine, ABC = abacavir, ART = antiretroviral therapy, BMD = bone mineral density (BMD).

Associations with low LS BMD at bivariate analysis are presented in **Table 3**. Females were more likely to have low LS BMD compared to males (cOR = 2.8, p = 0.04). Older age was also associated with low LS BMD (cOR = 1.38 per year older p = 0.001), as was older age at first line ART initiation (cOR = 1.17, p = 0.037).

**Table 3:** Bivariate Lumbar spine bone mineral density analysis, N = 159.

Characteristics	Abnormal	Normal	cOR	95%CI	P-Value
	N (%)	N (%)			
**Sex**					
Male	6 (7.59)	73 (92.41)			
Female	15 (18.75)	65 (81.25)	2.8	1.03–7.67	0.04
**WHO stage**					
Stage 1&2	19 (13.19)	125 (86.81)	1		
Stage 3&4	2 (13.3)	13 (86.67)	1.01	0.21–4.84	0.988
**Regimen**					
ABC- based	7 (9.33)	68 (90.7)	1		
ZDV- based	21 (25)	63 (75)	1.94	0.74–5.11	0.18
EFV- based	11 (13.75)	69 (86.25)	1		
NVP- based	10(12.7)	69(87.34)	0.91	0.36–2.27	0.84
	**Median (IQR)**	**Median (IQR)**			
Age	13 (11,14)	9.5 (7.0,12.0)	1.38	1.14–1.68	0.001
BMIZ	-2.0 (-2.0, -1.0)	-1.0 (-2.0, -1.0)	0.80	0.54–1.19	0.277
Weightz	-2.0 (2.0)	-1.0 (2.0)	1.09	0.78–1.55	0.591
Abs CD4	808 (615,970)	770 (505,1083)	1.00	0.99–1.00	0.782
CD4%	32 (26,37)	30 (22,37)	1.025	0.98–1.07	0.251
HIV RNA	3.97(3.49,4.54)	4.54(3.85,4.886)	0.56	0.30–1.05	0.07
Time on first-line ART	6.0 (5.0,8.0)	5.0 (3.0,7.0)	1.149	0.96–1.37	0.119
Age at first-line ART start	6.0 (4.0,8.0)	4.0 (2.0,6.0)	1.17	1.01–1.37	0.037

Abbreviations: IQR = interquartile range, BMIZ = body mass index Z-score, Weightz = weight Z score, HIV RNA = HIV viral load, ART = anti-retroviral therapy, EFV = efavirenz, NVP = nevirapine, ABC = abacavir, ZDV = zidovudine

The results of multivariate analysis are summarized in **Table 4**.

**Table 4:** Multivariate analysis of baseline characteristics and bone mineral density, N = 159.

**TBLH BMD**			
**Characteristic**	**aOR**	**95%CI**	**P-Value**
Age (/year older)	1.37	1.13–1.65	0.001
Female sex	3.80	1.31–10.81	0.014
ZDV compared to ABC based regimen	3.68	1.2510.80	0.018
BMIZ (/unit increase)	0.36	0.21–0.61	<0.001
**LS BMD**			
Age (/year older)	1.42	1.16–1.74	0.001
Female Sex	3.41	1.18–9.8	0.023

Abbreviations: aOR = adjusted odds ratio, BMIZ = body mass index Z-score, LS BMD = lumbar spine bone mineral density, TBLH BMD = total body less head bone mineral density, ZDV = zidovudine, ABC = abacavir

In the multivariate analysis of TBLH BMD, first-line ZDV exposure (aOR = 3.68, p = 0.018), older age (aOR = 1.37 per year older, p = 0.001), female sex (aOR = 3.8, p = 0.014) and lower BMI (aOR = 0.36 per unit higher, p<0.001) were independently associated with low TBLH BMD.

Females were more likely to have a lower LS BMD compared to males (aOR = 3.41, p = 0.023), and older age (aOR = 1.42 per year older, p<0.001) was independently associated with low LS BMD.

## Discussion

The overall prevalence of low BMD in our study was high at 21%, with higher detection by TBLH (18%) compared to the LS (13%), whereas 9% children had both. The factors associated with low TBLH BMD were age, sex and low BMI, which is consistent with the results of other studies. In our study, ZDV exposure was the only drug-related factor associated with low BMD. However, since this was a cross-sectional study, associations do not imply causality.

The prevalence of low BMD in this study is comparable to that in other studies that have described a high prevalence of low BMD in CLWH [8, 19]. However, a prevalence rate as low as 4–7% has been reported in well-resourced countries [20–22]. Associated inadequate nutrition may have contributed to the higher prevalence of low BMD, given that our study population resides in a resource-limited setting, where balanced diets are not readily available [23]. In addition, half of the participants had prolonged exposure to efavirenz (EFV), which disrupts calcium and vitamin D metabolism, that are essential for bone development [24]. Low vitamin D status has been described among CLWH in Africa with a prevalence of 17.3% [25]. Although it was not assessed in this study, a recent study among Ugandan CLWH found an association between EFV use and vitamin D deficiency [26].

Increasing age was independently associated with low BMD, which may be explained by the delayed puberty that has been observed in CLWH and is associated with delayed skeletal growth [19, 27]. More females in this study had low BMD compared to males in both TBLH and LS which is consistent with other studies [8]. Sex differences in pre-pubertal BMD are conflicting. However, females tend to have smaller bones compared to males. BMD is a two- dimensional measurement that tends to underestimate the BMD for smaller bones [28]. The females in the study population may have smaller bones compared to the reference population that had height- for- age Z-scores ranging from 0.2 to 0.45 [29].

Low BMD was associated with low BMI in this study similar to the results of other studies where adequate BMI- for- age was correlated with increased bone mass. Bones adapt to mechanical loading and an increase in load results in strengthening of bone by remodeling itself to accommodate the increased load, while a decrease in load causes bone weakening [6, 30, 31].

Data regarding the effect of ART on bone are variable. In previous studies, use of ZDV or EFV was associated with low BMD, which is consistent with our findings where low BMD was associated with ZDV use. Kim et al found bone loss in Korean adults who were exposed to ZDV for over a year [32]. Similarly in a longitudinal study, patients who switched to ZDV had a small but significant decrease in BMD compared to those taking ABC who remained stable [33]. This is also consistent with the in vitro observation that ZDV stimulates osteoclast activity in mice [34]. Paediatric data regarding ZDV and bone density is limited, although Bunders et al found no association between ZDV and low spinal BMD. Similarly, there was no association of ZDV with spinal BMD in our study [20]. There was also no significant association between EFV and BMD in our study. Adult studies have associated EFV with reduced BMD but there is limited data regarding BMD and EFV in the paediatric population [35]. However there was bone accrual in South African children who switched from lopinavir/ritonavir based ART to EFV [36]. This finding may have been due to change to a drug with a better bone safety profile but is difficult to compare with our study findings. Bunders et al found no association between low BMD and EFV in children which is similar to our findings [13, 19, 29, 30, 32, 33]. The mechanism for no association is unclear but may be due to the difference in the pharmacokinetics of EFV in children that are distinct from adults. Plasma clearance of EFV in children is faster than in adults and hence may have a less significant effect on bone health [37, 38].

The mechanisms through which HIV causes low BMD include osteoclast activation by the HIV viral protein gp120 and production of pro-inflammatory cytokine TNFα and interleukin 6 that increase bone resorption. There was no association between HIV viral load and BMD in our study. Viral load at a single time point may not be an accurate measure of lifetime HIV virological control. Different studies have reported inconsistent results. Dimeglio et al did not adjust for height hence they found that a high viral load was associated with low LS BMD. However, other paediatric studies found no association after height adjustment for BMD was done similar in our study where [21, 39, 40] height adjustment for BMD Z- score was performed.

There are several potential limitations of this study. The DXA scanner does not measure volumetric density but only measures areal density, therefore underestimates the BMD for smaller bones.

There are no local or African BMD reference data for children. The reference data used for determining BMD Z scores were obtained from the BMDCS that used a healthy black American population. It is possible that we may have overestimated low BMD due to the geographical, environmental and dietary differences between our study population and the reference data [15]. Thirdly there was no HIV negative control group from a similar environment. This was a single-site study which limits the generalizability.

## Conclusion

Low BMD is prevalent among Ugandan CLWH with virologic failure following first-line ART. Low TBLH BMD was associated with older age, female sex, low BMI and first- line ZDV exposure.

Health intervention programs should promote improved nutrition and also minimize the use of regimens associated with low BMD to prevent potential future fracture risk in low-income settings. Follow-up of these children in the CHAPAS-4 trial to a minimum of 96 weeks will enable us to evaluate the long-term effects of second-line ART on BMD. Future research should focus on therapeutic and preventive interventions and on monitoring the impact of these findings on bone health following transition to adult care.

## References

[1] Fact sheet—Latest global and regional statistics on the status of the AIDS epidemic.

[2] WHO | Consolidated guidelines on the use of antiretroviral drugs for treating and preventing HIV infection. WHO.

[3] Wren TAL, Kalkwarf HJ, Zemel BS, Lappe JM, Oberfield S, Shepherd JA, et al. Longitudinal Tracking of DXA Bone Measures Over 6 Years in Children and Adolescents: Persistence of Low Bone Mass to Maturity. The Journal of Pediatrics. 2014;164(6):1280–5.e2. 24485819 10.1016/j.jpeds.2013.12.040 PMC4035430

[4] Kruger MJ, Nell TA. Bone mineral density in people living with HIV: a narrative review of the literature. AIDS Res Ther. 2017;14(1):35. doi: 10.1186/s12981-017-0162-y 28747190 PMC5530558

[5] Eckard AR, Mora S. Bone Health in HIV-infected Children and Adolescents. Curr Opin HIV AIDS. 2016;11(3):294–300. doi: 10.1097/COH.0000000000000270 26890208 PMC4860809

[6] Mwaka ES, Munabi IG, Castelnuovo B, Kaimal A, Kasozi W, Kambugu A, et al. Low bone mass in people living with HIV on long-term anti-retroviral therapy: A single center study in Uganda. PLOS ONE. 2021;16(2):e0246389. doi: 10.1371/journal.pone.0246389 33544754 PMC7864439

[7] Katzenstein TL, Wessman M, Moseholm E, Sandholdt H, Hansen A-BE, Lebech A-M, et al. Prevalence of low bone mineral density among people living with HIV. Cogent Medicine. 2021;8(1):1920667.

[8] Rukuni R, Rehman AM, Mukwasi-Kahari C, Madanhire T, Kowo-Nyakoko F, McHugh G, et al. Effect of HIV infection on growth and bone density in peripubertal children in the era of antiretroviral therapy: a cross-sectional study in Zimbabwe. Lancet Child Adolesc Health. 2021;5(8):569–81. doi: 10.1016/S2352-4642(21)00133-4 34139202 PMC8295041

[9] Bhise S, Jain A, Savardekar L, Shetty N, Shah I. Bone health in HIV-infected children on antiretroviral therapy: An Indian study. Indian J Sex Transm Dis. 2021;42(2):138. doi: 10.4103/ijstd.IJSTD_35_19 34909618 PMC8628105

[10] Arpadi SM, Shiau S, Marx-Arpadi C, Yin MT. Bone health in HIV-infected children, adolescents and young adults: a systematic review. J AIDS Clin Res. 2014;5(11). doi: 10.4172/2155-6113.1000374 26504618 PMC4618404

[11] Hoy JF, Grund B, Roediger M, Schwartz AV, Shepherd J, Avihingsanon A, et al. Immediate Initiation of Antiretroviral Therapy for HIV Infection Accelerates Bone Loss Relative to Deferring Therapy: Findings from the START Bone Mineral Density Substudy, a Randomized Trial. Journal of bone and mineral research: the official journal of the American Society for Bone and Mineral Research. 2017;32(9):1945. doi: 10.1002/jbmr.3183 28650589 PMC5555813

[12] Brown TT, McComsey GA, King MS, Qaqish RB, Bernstein BM, da Silva BA. Loss of Bone Mineral Density After Antiretroviral Therapy Initiation, Independent of Antiretroviral Regimen. JAIDS Journal of Acquired Immune Deficiency Syndromes. 2009;51(5):554–61. doi: 10.1097/QAI.0b013e3181adce44 19512937

[13] Chisati EM, Constantinou D, Lampiao F. Reduced bone mineral density among HIV infected patients on anti-retroviral therapy in Blantyre, Malawi: Prevalence and associated factors. PLOS ONE. 2020;15(1):e0227893. doi: 10.1371/journal.pone.0227893 31935270 PMC6959680

[14] Couffignal C, Kolta S, Flamant M, Cazanave C, Haymann J-P, Mentré F, et al. Nevirapine Use Is Associated with Higher Bone Mineral Density in HIV-1 Positive Subjects on Long-Term Antiretroviral Therapy. AIDS Research and Human Retroviruses. 2020;36(5):399–405. doi: 10.1089/AID.2019.0229 31891665

[15] Zemel BS, Kalkwarf HJ, Gilsanz V, Lappe JM, Oberfield S, Shepherd JA, et al. Revised Reference Curves for Bone Mineral Content and Areal Bone Mineral Density According to Age and Sex for Black and Non-Black Children: Results of the Bone Mineral Density in Childhood Study. J Clin Endocrinol Metab. 2011;96(10):3160–9. doi: 10.1210/jc.2011-1111 21917867 PMC3200252

[16] World Health O. WHO case definitions of HIV for surveillance and revised clinical staging and immunological classification of HIV-related disease in adults and children. 2007:48.

[17] Cole TJ, Freeman JV, Preece MA. British 1990 growth reference centiles for weight, height, body mass index and head circumference fitted by maximum penalized likelihood. Stat Med. 1998;17(4):407–29. 9496720

[18] ISCD [Internet]. Available from: https://iscd.org/learn/official-positions/pediatric-positions/files/820/pediatric-positions.html.

[19] Gregson CL, Hartley A, Majonga E, McHugh G, Crabtree N, Rukuni R, et al. Older age at initiation of antiretroviral therapy predicts low bone mineral density in children with perinatally-infected HIV in Zimbabwe. Bone. 2019;125:96–102. doi: 10.1016/j.bone.2019.05.012 31082498 PMC6599174

[20] Bunders MJ, Frinking O, Scherpbier HJ, van Arnhem LA, van Eck-Smit BL, Kuijpers TW, et al. Bone Mineral Density Increases in HIV-Infected Children Treated With Long-term Combination Antiretroviral Therapy. Clinical Infectious Diseases. 2013;56(4):583–6. doi: 10.1093/cid/cis917 23097583

[21] Dimeglio LA, Wang J, Siberry GK, Miller TL, Geffner ME, Hazra R, et al. Bone Mineral Density in Children and Adolescents with Perinatal HIV Infection. AIDS (London, England). 2013;27(2):211–20. doi: 10.1097/QAD.0b013e32835a9b80 23032412 PMC4157938

[22] Atencio P, Cabello A, Conesa-Buendía FM, Pérez-Tanoira R, Prieto-Pérez L, Carrillo I, et al. Increased risk factors associated with lower BMD in antiretroviral-therapy–naïve HIV-infected adult male. BMC Infect Dis. 2021;21(1):542. 34107907 10.1186/s12879-021-06263-9 PMC8188666

[23] Abate BB, Aragie TG, Tesfaw G. Magnitude of underweight, wasting and stunting among HIV positive children in East Africa: A systematic review and meta-analysis. PLOS ONE. 2020;15(9):e0238403. doi: 10.1371/journal.pone.0238403 32941443 PMC7498078

[24] Theodorou M, Sersté T, Gossum MV, Dewit S. Factors associated with vitamin D deficiency in a population of 2044 HIV-infected patients. Clinical Nutrition. 2014;33(2):274–9. doi: 10.1016/j.clnu.2013.04.018 23680190

[25] Mogire RM, Morovat A, Muriuki JM, Mentzer AJ, Webb EL, Kimita W, et al. Prevalence and predictors of vitamin D deficiency in young African children. BMC Med. 2021;19:115. doi: 10.1186/s12916-021-01985-8 34011341 PMC8136043

[26] Piloya TW, Bakeera–Kitaka S, Kisitu GP, Idro R, Cusick SE. Vitamin D status and associated factors among HIV-infected children and adolescents on antiretroviral therapy in Kampala, Uganda. PLOS ONE. 2021;16(6):e0253689. doi: 10.1371/journal.pone.0253689 34166428 PMC8224887

[27] Bellavia A, Williams PL, Dimeglio LA, Hazra R, Abzug MJ, Patel K, et al. Delay in Sexual Maturation in Perinatally HIV-Infected Youth Is Mediated by Poor Growth. AIDS (London, England). 2017;31(9):1333–41. 28358737 10.1097/QAD.0000000000001486 PMC5472204

[28] Binkovitz LA, Henwood MJ. Pediatric DXA: technique and interpretation. Pediatr Radiol. 2007;37(1):21–31. doi: 10.1007/s00247-006-0153-y 16715219 PMC1764599

[29] Kalkwarf HJ, Zemel BS, Gilsanz V, Lappe JM, Horlick M, Oberfield S, et al. The Bone Mineral Density in Childhood Study: Bone Mineral Content and Density According to Age, Sex, and Race. J Clin Endocrinol Metab. 2007;92(6):2087–99. doi: 10.1210/jc.2006-2553 17311856

[30] Palchetti CZ, Szejnfeld VL, de Menezes Succi RC, Patin RV, Teixeira PF, Machado DM, et al. Impaired bone mineral accrual in prepubertal HIV-infected children: a cohort study. Braz J Infect Dis. 2015;19(6):623–30. doi: 10.1016/j.bjid.2015.08.010 26477385 PMC9425359

[31] Rowe P, Koller A, Sharma S. Physiology, Bone Remodeling. StatPearls. Treasure Island (FL): StatPearls Publishing; 2022. 29763038

[32] Kim H-S, Chin BS, Shin H-S. Prevalence and Risk Factors of Low Bone Mineral Density in Korean HIV-Infected Patients: Impact of Abacavir and Zidovudine. J Korean Med Sci. 2013;28(6):827–32. doi: 10.3346/jkms.2013.28.6.827 23772145 PMC3677997

[33] Martin A, Smith DE, Carr A, Ringland C, Amin J, Emery S, et al. Reversibility of lipoatrophy in HIV-infected patients 2 years after switching from a thymidine analogue to abacavir: the MITOX Extension Study. AIDS. 2004;18(7):1029. doi: 10.1097/00002030-200404300-00011 15096806

[34] Pan G, Kilby M, McDonald JM. Modulation of Osteoclastogenesis Induced by Nucleoside Reverse Transcriptase Inhibitors. AIDS Research and Human Retroviruses. 2006;22(11):1131–41. doi: 10.1089/aid.2006.22.1131 17147500 PMC1994207

[35] Dave JA, Cohen K, Micklesfield LK, Maartens G, Levitt NS. Antiretroviral Therapy, Especially Efavirenz, Is Associated with Low Bone Mineral Density in HIV-Infected South Africans. PLOS ONE. 2015;10(12):e0144286. doi: 10.1371/journal.pone.0144286 26633015 PMC4669137

[36] Arpadi SM, Shiau S, Strehlau R, Patel F, Mbete N, McMahon DJ, et al. Efavirenz is associated with higher bone mass in South African children with HIV. AIDS (London, England). 2016;30(16):2459–67. doi: 10.1097/QAD.0000000000001204 27427876 PMC5069169

[37] Fillekes Q, Natukunda E, Balungi J, Kendall L, Bwakura-Dangarembizi M, Keishanyu R, et al. Pediatric Underdosing of Efavirenz: A Pharmacokinetic Study in Uganda. JAIDS Journal of Acquired Immune Deficiency Syndromes. 2011;58(4):392–8. doi: 10.1097/QAI.0b013e318235e560 21926634

[38] Fletcher CV, Brundage RC, Fenton T, Alvero CG, Powell C, Mofenson LM, et al. Pharmacokinetics and Pharmacodynamics of Efavirenz and Nelfinavir in HIV-infected Children Participating in an Area-under-the-curve Controlled Trial. Clin Pharmacol Ther. 2008;83(2):300–6. doi: 10.1038/sj.clpt.6100282 17609682 PMC2848440

[39] Mahtab S, Scott C, Asafu-Agyei NAA, Machemedze T, Frigati L, Myer L, et al. Prevalence and predictors of bone health among perinatally HIV-infected adolescents. AIDS (London, England). 2020;34(14):2061–70. doi: 10.1097/QAD.0000000000002686 32910060 PMC7606491

[40] Puthanakit T, Saksawad R, Bunupuradah T, Wittawatmongkol O, Chuanjaroen T, Ubolyam S, et al. Prevalence and risk factors of low bone mineral density among perinatally HIV-infected Thai adolescents receiving antiretroviral therapy. J Acquir Immune Defic Syndr. 2012;61(4):477–83. doi: 10.1097/QAI.0b013e31826ea89b 22918157 PMC3518550

