# Correction: Bone mineral density among children living with HIV failing first-line anti-retroviral therapy in Uganda: A sub-study of the CHAPAS-4 trial

**DOI:** 10.1371/journal.pone.0339209

**Published:** 2025-12-16

**Authors:** Eva Natukunda, Alex Szubert, Caroline Otike, Imerida Namyalo, Esther Nambi, Alasdair Bamford, Katja Doerholt, Diana M. Gibb, Victor Musiime, Phillipa Musoke

The Funding statement for this article is incorrect. The correct Funding statement is as follows: Funded by the European and Developing Countries Clinical Trials Partnership (EDCTP) as part of the EDCTP programme, which is supported by the European Union through a grant (TRIA20151078) awarded to DMG.
