# Supplementary material for: Bone mineral density among children living with HIV failing first-line anti-retroviral therapy in Uganda: A sub-study of the CHAPAS-4 trial
Source: PLoS One. 2023 Jul 20;18(7):e0288877. doi: 10.1371/journal.pone.0288877 (PMC10359007; doi:10.1371/journal.pone.0288877)
Supplement: Attachment — Submitted filename: Response to reviewers.docx [file pone.0288877.s001.docx]

**Response to reviewers’ comments**

- A rebuttal letter that responds to each point raised by the academic editor and reviewer(s). You should upload this letter as a separate file labeled 'Response to Reviewers'.

**Response: Rebuttal letter has been uploaded**

- A marked-up copy of your manuscript that highlights changes made to the original version. You should upload this as a separate file labeled 'Revised Manuscript with Track Changes'.

**Response: Revised manuscript with track changes has been uploaded**

- An unmarked version of your revised paper without tracked changes. You should upload this as a separate file labeled 'Manuscript'.

**Response: Revised paper without tracked changes has been uploaded**

● The name of the colleague or the details of the professional service that edited your manuscript

**Response: The editing company is editage**
● A copy of your manuscript showing your changes by either highlighting them or using track changes (uploaded as a *supporting information* file)

**Response-A copy with track changes has been uploaded**
● A clean copy of the edited manuscript (uploaded as the new *manuscript* file)

- 3. We note that the grant information you provided in the ‘Funding Information’ and ‘Financial Disclosure’ sections do not match.
- When you resubmit, please ensure that you provide the correct grant numbers for the awards you received for your study in the ‘Funding Information’ section.

**Response: 1.Funding source was European and Developing Countries Clinical Trials Partership. Grant number TRIA2015-1078**

**2. Janssen Pharmaceuticals**

**3.Viatris**

- 4. Thank you for stating the following in the Acknowledgments Section of your manuscript: 
  "This work was done as part of the CHAPAS 4 trial. The authors would like to thank the children and their caretakers who volunteered to participate in this study.
  We thank all the data collection team at the Joint Clinical Research Centre Kampala
  CHAPAS 4 trial is mainly funded by the European and Developing Countries Clinical Trials Partnership (grant number TRIA2015-1078) and Medical Research Council (MRC)
  Additional support was received from: Gilead Sciences, Janssen Pharmaceuticals, ViiV Health care and CIPLA"
- We note that you have provided funding information that is not currently declared in your Funding Statement. However, funding information should not appear in the Acknowledgments section or other areas of your manuscript. We will only publish funding information present in the Funding Statement section of the online submission form.

**Response: Funding information has been deleted from the manuscript**
Please remove any funding-related text from the manuscript and let us know how you would like to update your Funding Statement. Currently, your Funding Statement reads as follows: 
"DMG received the award from European and Developing Countries Clinical Trials Partnership (EDCTP) grant number TRIA2015-1078.https://www.edctp.org
Additional support was provided by Gilead sciences , https://www.gilead.com,Jannsen Pharmaceuticals,https://www.janssen.comViiV Health care,https://viivhealthcare.com
The sponsors had no role in study design,data collection and analysis,decision to publish or preparation of the manuscript"

- Please include your amended statements within your cover letter; we will change the online submission form on your behalf.

**Response: Ammended statement 1. DMG received the award from European and Developing Countries Clinical Trials Partnership (EDCTP) grant number TRIA2015-1078.https://www.edctp.org**

**2. DMG received funding from Jannsen Pharmaceuticals,** [**https://www.janssen.com**](https://www.janssen.com)

**3. DMG received funding from Viatris, https://www.viatris.com
The sponsors had no role in study design,data collection and analysis,decision to publish or preparation of the manuscript"**

- 5. We note that you have stated that you will provide repository information for your data at acceptance. Should your manuscript be accepted for publication, we will hold it until you provide the relevant accession numbers or DOIs necessary to access your data. If you wish to make changes to your Data Availability statement, please describe these changes in your cover letter and we will update your Data Availability statement to reflect the information you provide.

**Response: There are no changes to the data availability statement**
[Note: HTML markup is below. Please do not edit.]

Reviewers' comments:

- Please use the space provided to explain your answers to the questions above. You may also include additional comments for the author, including concerns about dual publication, research ethics, or publication ethics. (Please upload your review as an attachment if it exceeds 20,000 characters)
- Reviewer #1: This is an interesting study that highlights the prevalence of co-morbidities such as low BMD in CLWH, especially pertaining to the cohort that is switching treatment due to virologic failure.
  These observations are even more relevant in populations that are resource-limited and do not have access to a balanced diet.
  The manuscript if re-structured in the context of the larger question addressed in the CHAPAS4 trial will have significant implications.
  1. Although this study does an excellent job characterizing the BMD, LSBMD, and TBLH in CLWH, it is unclear how these findings address the questions asked in the CHAPAS4 trial and specifically pertaining to the toxicity sub-study.

**Response: This manuscript describes baseline bone health status as a benchmark against which the bone outcomes can be compared. Subsequent manuscripts are underway to describe the longitudinal data that describes the toxicities associated with the trial drugs of which bone loss is among.**
2. The authors have not been able to tease out the impact of the high viral load and the first-line ART (BAC-based, ZDV based, etc.) on BMD.

**Response: Thank you for this observation, the authors did explore the impact of high viral load and the first-line ART (ABC-based, ZDV based, etc.) on BMD. However, there was no statistically significant association between viral load, first line ART and BMD which is reflected in the results and the discussion.**
3. Did the authors normalize or control for low BMI as a confounder to low BMD, since the study was conducted in a low-resource environment?

**Response:** **The authors included BMI in the variable list and assessed for confounding during analysis, with a relative difference between the crude and adjusted OR being 10% or more considered as confounding.**
4. It is unclear from the results and the lack of description in the manuscript, the characteristics (if any) of the age-matched controls or if the "normal" BMD is that of controls.

**Response: Thank you very much,there were no controls in this study.**
5. There should be 2 separate control cohorts (if feasible) included in this study
a. Uninfected controls with normal BMD
b. CLWH that responded to the first line of ART

**Response: Collecting this information was not feasible**
6. There is no graphical representation of the data.

**Response: According to study objectives, the most suitable data presentation format was tables. Hence, we considered tables in this manuscript.**

7. Details of the DXA and parameters of the scans and the analyses need to be included. Was the same DXA machine used for performing scans on all participants?

**Response:** **One DXA machine was used for performing all the scans.**
8. The interquartile range is a useful measure of statistical dispersion because it is less sensitive to outliers. It will be useful to compare the median to that of control subjects to identify any potential outliers.

**Response: In this study, we only collected data for CLWH who were failing on ART and did not have a control population**

- Reviewer #2: Thank you for a well written paper on the prevalence and associated factors of low BMD in your study population of children switching to second line ART due to virological failure. The findings showed a high prevalence of low BMD and several associated factors, including age, sex, BMI and AZT use. I think highlighting the need for following up these children/adolescents as they transition into adult care and assessing the long term outcomes, in terms of possible fracture risk, is very important as this is not a risk that is frequently monitored/assessed in the resource-limited settings where these adolescents are being cared for. I recommend that the article is accepted for publication after minor revisions.
  Major Revisions
  Nil
  Minor Revisions
  Abstract Results: Please review minor grammar, capitals and spaces before/after brackets as needed
  Abstract Conclusion: “and development of cost-effective screening tools to identify poor bone health in HIV+ adolescents entering adulthood.” Development of screening tools is not mentioned in your main text. Either add this into your main text (because it is very important) or adjust your abstract conclusion to reflect your main text conclusion

  Introduction: “Because of early ART initiation…” Suggest rephrasing this sentence so that it does not begin with “Because”

**Response: The text has been corrected**

Introduction: “ART-related comorbidities including low bone mineral density (BMD) are becoming…” Suggest adding commas to read “comorbidities, including low bone mineral density (BMD), …”
**Response: The text has been updated**
Introduction: “where the majority of children” Should this read “where the majority of CLWH reside”
**Response: Updated**
Introduction: “and where malnutrition that also contributes to low BMD is common” Suggest adding commas to read “and where malnutrition, which also contributes to low BMD, is common”

**Response: Updated**

Introduction: “also contributes to low BMD is common. [8, 10-12] However BMD
is higher in the black population compared” The link between these two sentences needs to be more clearly explained

**Response:The text has been updated**

Introduction: “zidovudine (ZDV), contribute to” Suggest removing comma here
**Response: The text was Updated**
Introduction “also been documented to be associated” Suggest removing “as documented” from this sentence to make it easier to read
**Response: Updated to “has been found”**
Introduction: “BMD is determined by z-scores in adolescents and children according to the International Society for Clinical Densitometry 2019(ISCD).[16] Low BMD is defined as z--scores less or equal to -2 defines low BMD.[16]. Suggest moving this paragraph to methods. The second sentence of this paragraph has repetition and needs to be reviewed.

**Response: The sentence has been moved to methods**

Methods: “lamivudine (ZDV/3TC), depending on first-line). Children” Suggest removing bracket at the end of the sentence.

**Response: Bracket removed**

Methods: “Zimbabwe and Uganda were consecutively enrolled” Should this say simultaneously instead of consecutively?

**Response: The text has been updated to simultaneously**

Methods: “Reference BMD Z-scores were available for children who were ≥ 5 years.” If this is the reason only children > 5 were enrolled suggest making this clearer

**Response: Added “And therefore limited the analysis for children who were 5 years and above “**

Methods: “and classification of HIV disease [18], first” Please clarify what classification was used in the text

**Response:Updated to WHO classification**

Methods: “The LS BMD assesses trabecular” This is the first time this abbreviation is used, please put lumbar spine (LS)
**Response:Abbreviation added**
Methods: “children aged eight years and above gave written informed assent according to and as appropriate based on, knowledge of HIV status.” This sentence is not clear, please review (possibly remove the comma and say knowledge of their HIV status)
**Response:Sentence revised**
Methods: “or (LS) BMD z-score less than or equal to -2.” Remove brackets around LS
**Response: Brackets removed**
Results: “with only 15(9.45%) ever had stage 3 and 4.” Suggest changing to “ever having”

**Response: Changed to “ever having”**

Results: “Associations with low total body less head (TBLH) BMD are summarised” Can just use TBLH here as abbreviation explained previously in the text. It will make it easier to read

**Response: Updated**

Results: “Increasing age was associated with low TBLH BMD (cOR=1.33 per year older=0.001). Low BMI was also associated with low BMD (cOR=0.43 per unit increase. p<0.001).” Suggest making this into one sentence.
**Response: Updated**
Results: “Associations with low lumbar spine (LS) BMD” Can just use LS here as abbreviation explained previously

**Response: Abbreviation inserted**

Results: “LSBMD” separate into LS BMD throughout text

**Response:Updated**

Results: “Multivariate analysis is summarized in Table 4,” Is this part of the next paragraph? Not clear
**Response: Paragraph was formatted**
All tables: all abbreviations used in the table must be shown below the table, including ARVs. Please check this with all your tables

**Response:Updated**

Discussion: “Paediatric data regarding ZDV and bone density is limited though in one study bunders et al found no association with spinal BMD.Similarly,there was no association with spinal BMD in our study [22]” Capitalise Bunders. Please clarify these two sentences…no association with low or worsening BMD?
**Response: Bunders was capitalised**
Discussion: “HIV viral protein gp120 causes osteoclast activation while pro-inflammatory cytokine TNFα and interleukin 6 increase bone resorption. There was no association between HIV viral load and BMD in our study. Viral load at a single time point may not be an accurate measure of lifetime HIV virological control. Different studies reported inconsistent results. Dimeglio et al found high viral load was associated with low LSBMD while other paediatric studies found no association after height adjustment for BMD was done as in our study. In our study height adjustment for BMD Z- score was done.[23, 41, 42]
It would be useful to link the first sentence into the rest of the paragraph more, by making it clear that those are potential ways HIV causes low BMD. Did Dimeglio et al not adjust for height and that is why they potentially found a low LSBMD compared to other studies that did height adjust? This is not clear in this paragraph. Your references at the end should be after the second to last sentence where the studies are mentioned.
**Response-The text has been revised to link the sentences and also references moved**
Discussion: “Thirdly there was no control group from a similar environment.” Suggest adding in what sort of control group may have improved the study…HIV negative children from the same environment? HIV positive children who are virally suppressed?

**Response: This information has been included in the limitations**

Discussion: “And this was a single- site study limiting generalizability.” Please rephrase so that the sentence does not begin with And.

- **Response-Sentence has been revised**

  Discussion: “Vitamin D is essential for bone formation. Low Vitamin D status has been described among children living in Africa with a prevalence of 17.3%.[43] We did not assess vitamin D status but our study participants were exposed to EFV that may interfere with vitamin D metabolism.” This is already mentioned in the second paragraph of your discussion. Suggest merging this paragraph into that second paragraph. It is a bit repetitive and out of place after the limitations.
- **Response-The paragraph was revised and repeated statements deleted**

[
